# Molecular characterization of the lipophorin receptor in the crustacean ectoparasite *Lepeophtheirus salmonis*

**DOI:** 10.1371/journal.pone.0195783

**Published:** 2018-04-12

**Authors:** Muhammad Tanveer Khan, Sussie Dalvin, Qaiser Waheed, Frank Nilsen, Rune Male

**Affiliations:** 1 Sea Lice Research Centre, Department of Biological Sciences, University of Bergen, Bergen, Norway; 2 Sea Lice Research Centre, Institute of Marine Research, Bergen, Norway; 3 Computational Biology Unit, Department of Biological Sciences, University of Bergen, Bergen, Norway; Pusan National University, REPUBLIC OF KOREA

## Abstract

The Salmon louse (*Lepeophtheirus salmonis*) is a marine ectoparasite of salmonid fish in the Northern Hemisphere and considered as a major challenge in aquaculture and a threat to wild populations of salmonids. Adult female lice produce a large number of lipid-rich eggs, however, the mechanism of maternal lipid transport into developing eggs during salmon louse reproduction has not been described. In the present study, a full-length *L*. *salmonis* lipophorin receptor (*LsLpR*) consisting of 16 exons was obtained by RACE and RT-PCR. The predicted ORF was 952 amino acids and structural analysis showed five functional domains that are similar to LpR of insects and decapods. Phylogenetic analysis placed the LsLpR together with LpRs from decapods and insects. Expression analysis revealed that the relative abundance of *LsLpR* transcripts was highest in the larvae and adult female lice. In adult females, the *LsLpR* transcripts and protein were found in the ovary and vitellogenic oocytes whereas, in larvae, the *LsLpR* transcripts were found in the neuronal somata of the brain and the intestine. Oil Red O stain results revealed that storage of neutral lipids was found in vitellogenic oocytes and ovaries of adult females, and in the yolk of larvae. Moreover, RNA interference (RNAi) was conducted to demonstrate the function of *LsLpR* in reproduction and lipid metabolism in *L*. *salmonis*. In larvae, the transcription of *LsLpR* was decreased by 44–54% while in an experiment *LsLpR* knockdown female lice produced 72% less offspring than control lice.

## Introduction

The salmon louse (*Lepeophtheirus salmonis*) is a marine ectoparasitic copepod that infests salmonids in Norway, Scotland, Ireland and Canada. It feeds on blood, mucus and skin of hosts in sea water, which leads to major health and welfare issues of fish and results in a major economic losses in the Atlantic salmon (*Salmo Salar*) farming industry [1]. The salmon louse has also been considered to be a threat to wild salmonids [2]. The life cycle of the salmon louse comprises of eight developmental stages, each stage separated by a moult [3]. The free-living stages consist of two nauplius stages, and an infectious copepodid stage. After the settlement of copepodids to host fish, there are two immobile chalimus stages where the louse is anchored to the host through frontal filaments, followed by three mobile stages: two pre-adult stages and one adult stage. Eggs hatch into free-living nauplius I larvae, the first of three larval stages of *L*. *salmonis* that are lecithotrophic. These larvae stages rely on stored nutrients imported to the eggs during vitellogenesis and the free-living copepodids must settle to a fish host before they run out of energy [4].

Once the adult female louse becomes sexually mature, a continuous production of eggs is initiated in the ovaries. The oocytes migrate from the ovaries to the genital segment where they grow and mature forming two genital complexes with vitellogenic oocytes. The eggs are fertilized and deposited as a pair of egg-strings which the female carry externally until all eggs are hatched. Like other oviparous animals, salmon lice store large amounts of yolk proteins [5, 6] and lipids [7] in the developing oocytes to secure energy for embryogenesis and early larval development. In general, yolk lipids provide energy, building blocks for the developing cell membranes, and precursors for prostaglandin and steroid hormones. The major neutral lipid found in eggs and larvae (nauplius II) of *L*. *salmonis* is triacylglycerol (TAG), whereas the major polar lipids are phosphatidylcholine and phosphatidylethanolamine [7]. Despite the existing knowledge of lipid classes in oocytes and larvae of *L*. *salmonis*, mechanism of lipid distribution and uptake in developing oocytes is scarce. Hence, improved understanding of mechanism for lipid uptake will enhance the knowledge regarding oocytes maturation and can potentially be used in anti-parasitic strategies.

In animals, lipids are transported in the aqueous environment of the circulatory system in lipid-protein complexes named lipoproteins. A lipoprotein particle consists of a hydrophobic core of neutral lipids surrounded by a single layer of phospholipid molecules, unesterified cholesterol and apolipoproteins. Mammals have two different TAG-rich lipoproteins involved in lipid transport: chylomicrons from the intestine and very low-density lipoproteins (VLDL) from the liver, delivering neutral lipids to target tissues through lipoprotein lipase-mediated lipolysis. After lipolysis, chylomicrons convert into chylomicron remnants and VLDLs change into intermediate-density lipoproteins (IDLs) and low-density lipoproteins (LDLs). These remnants particles become enriched in cholesteryl ester (CE) and supply cholesterol to the liver or peripheral tissues through receptor-mediated endocytosis. In contrast to mammals, the major lipoprotein in the hemolymph of insects is lipophorin (Lp) [8–10] which functions as a reusable shuttle for the delivery of lipids to various tissues including oocytes [11–16]. In some insects, Lp is accumulated inside the developing oocytes and becomes itself part of the yolk [17]. Two forms of Lp are found in insects, high-density lipophorin (HDLp) and low-density lipophorin (LDLp) which has 30–50% and up to 62% lipid contents respectively [18, 19]. The HDLp contains one molecule of apoLp I and one molecule of apoLp II. However, when large amounts of lipids are mobilised during insect flight, extra copies of apoLp III are associated with HDLp and formation of LDLp occurs which contains much more lipids than HDLp [10, 12, 20]. Other than Lp, a small contribution of vitellogenin (Vg) has also been suggested in the transport of lipids to growing oocytes of insects [15, 18].

The LDL receptor (LDLR) is a member of the LDLR superfamily. In mammals, LDLR binds cholesterol-rich LDL and internalizes it through receptor-mediated endocytosis. During endocytosis, the receptor releases lipoprotein into the lumen of the endosome and the receptor is recycled back to the surface of the cell available to new rounds of endocytic uptake [21–23]. The role of LDLR is to maintain the cholesterol homoeostasis and mutations in this receptor lead to familial hypercholesterolemia [24, 25]. Another member of the LDLR superfamily, termed VLDL/Vg receptor (VLDLR/VgR) plays a major role in reproduction of chicken as it mediates the uptake of VLDL and Vg in the developing oocytes [26]. In arthropods, the LDLR family member lipophorin receptor (LpR) binds and transport lipophorin to the developing oocytes through receptor-mediated endocytosis. The LpR gene was first characterized at the molecular and functional level in the locust, *Locusta migratoria* [27] and later cloned and characterized in several insect species [28–35]. Recently, three lipophorin receptors (LpR1, LpR2A and LpR2B) from shrimp (*Pandalopsis japonica*) have been characterised [36]. Similar to other members of LDLR family, LpR contains five functional domains: A ligand binding domain, an epidermal growth factor (EGF) precursor homologous domain, an O-linked sugar domain, a transmembrane domain and a cytoplasmic domain. In insects, the LpR has been reported to play an important function in lipid metabolism as well as in the reproduction. The expression of LpRs takes place predominantly in the reproductive organs and is responsible for lipid accumulation in growing oocytes. Studies of mutants have shown that LpR2 of *D*. *melanogaster* has an important role in the transport of lipids to growing oocytes [34]. Similarly, RNAi experiment showed that LpR is involved in the uptake of Lp in *B*. *germanica* [32].

In this study, a gene encoding a lipophorin receptor (LsLpR) containing the conserved domain structure was identified in salmon louse. To our knowledge, this is the first report on the characterization of a member of LDLR superfamily in *L*. *salmonis*. The receptor was found to be expressed in all developmental stages, but predominantly in larval and adult female lice. The receptor mRNA and protein were found exclusively in the ovaries and oocytes of the adult females. In larvae, the transcripts were found in several tissues. Furthermore, RNAi experiments were conducted in larvae and female lice confirming this function.

## Materials and methods

### Sampling of salmon lice

A laboratory strain of salmon lice, *Lepeophtheirus salmonis* [37] was maintained on Atlantic salmon (*Salmo salar*) in tanks, supplied with a continuous flow of seawater at 10°C and 34.5 ppt salinity. Fish were hand fed daily with commercial dry pellets. Nauplii I/II and free-living copepodids were obtained from egg-strings, hatched in flow-through incubators with the same supply of seawater. Chalimi, pre-adult and adult stages of lice were sampled from fish. Before sampling, fish were anaesthetized with a mixture of benzocaine (60mg/l) and methomidate (5mg/l) in seawater. All the experiments were performed according to the Norwegian animal welfare legislations and approved by Norwegian Food Safety Authority (Mattilsynet).

Five biological replicates were collected from each developmental stage of the salmon lice for stage-specific RT-qPCR. The following life stages and pooled number of animals were harvested for each replicate: Nauplius I (n = 100), nauplius II (n = 100), planktonic copepodid (n = 100), chalimus I (n = 10), chalimus II (n = 10), preadult I male and female (n = 1), preadult II male and female (n = 1), adult male (n = 1) and adult female (n = 1). All the samples were collected in RNAlater^TM^ (Ambion) and kept overnight at 4°C before long time storage at -20°C.

### Isolation of RNA and cDNA synthesis

Total RNA was isolated using TRI reagent (Sigma-Aldrich) as per manufacturer's instructions. The concentration and purity of isolated RNA was confirmed using Nanodrop ND-1000 spectrophotometer (NanoDrop Technologies). Following RNA isolation, 1 μg of total RNA was treated with amplification grade DNaseI (Invitrogen) as per manufacturer’s instructions. For RT-qPCR, 300 ng of total DNase-treated RNA was used for the synthesis of cDNA with Affinity Script QPCR cDNA Synthesis Kit (Stratagene) and diluted 10 times with nuclease free water prior to storage at -20°C. For RT-PCR, 1μg of total RNA was reverse transcribed using a qScript cDNA SuperMix (Quanta Bioscience).

### Genome analysis, PCR, cloning and sequencing of LsLpR

The LpR sequences from *Bombyx mori* (GenBank: AB211594) and *Blattella germanica* (GenBank: AM403063) were used to identify candidate LpR genes in the salmon louse genome database (Licebase https://licebase.org/). Two genes (stable IDs: EMLSAG00000008639 and EMLSAG00000009473) were predicted to encode LpRs according to the lowest e-value criteria. However, SMARTer RACE (rapid amplification of cDNA ends) demonstrated that these two predicted genes were part of the same gene. The 5′ and 3′ RACE was conducted with SMARTer RACE cDNA Amplification Kit (Clontech) as instructed in the users’ manual. Total RNA isolated from an adult female was used to synthesize the 5′ and 3′ RACE-Ready cDNAs using gene-specific primers (Table 1). PCR products were cloned into pCR™ 4-TOPO® vector using the TOPO TA Cloning kit for sequencing (Life Technologies) followed by transformation into *Escherichia coli* TOP10 cells. PCR products of positive clones were cleaned with ExoSAP-it (Affymetrix) and used as templates for sequencing using M13 forward and reverse primers. All the sequences were assembled, and the single transcript was reconfirmed by RT-PCR. The complete mRNA sequence of LsLpR has been deposited in GenBank (MF435899).

**Table 1:** Primers used during this study.

**Name**	**Sequence (5'-3')**	**Analysis**
LpR48_5RACE	CTCCACAATCATCCTCTTGATCACAAACCCAAC	RACE
LpR_3RACE-3	GCAAGGCATCAGAAGAAGGCAATGGATCTCG	RACE
LpR-F	TCCATCTCTTCTGTTTGCACAT	PCR
LpR-R	ACAACGATAGATCGCCATGA	PCR
LpR-F2	GCGTGTCTCAAGGGTCACAT	PCR
LpR-R2	CACGTCTGATCACATCCTCCA	PCR
M13_f	GTAAAACGACGGCCAG	TOPO cloning
M13_r	CAGGAAACAGCTATGAC	TOPO cloning
LpRORF-F	ATGATACGTTTCTCAACATA	PCR
LpRORF-R	CGAATTGATGACCTCCTCTGA	PCR
LpRp-F T7	TAATACGACTCACTATAGGGGCACCCATTGATGAAGGTAA	dsRNA, Fragment 1
LpRp2-R T7	TAATACGACTCACTATAGGGGATGACCATTGGGACTTGCT	
LpRp-F	GCACCCATTGATGAAGGTAA	
LpRp2-R	GATGACCATTGGGACTTGCT	
LpRp-FT7	TAATACGACTCACTATAGGGGAAACTGGGCGGATGAGTCA	dsRNA, Fragment 2, In situ
LpRp-RT7	TAATACGACTCACTATAGGGGTTCCCGTATCTGTCCAATA	
LpRp-F	GAAACTGGGCGGATGAGTCA	
LpRp-R	GTTCCCGTATCTGTCCAATAGA	
LpRp-F3 T7	GAAATTAATACGACTCACTATAGGGTAACGAGACTGCCGGATTCA	dsRNA Fragment 3
LpRp-R3 T7	GAAATTAATACGACTCACTATAGGGACAGCATGATCTCTTGGTTCAC	
LpRp-F3	TAACGAGACTGCCGGATTCA	
LpRp-R3	ACAGCATGATCTCTTGGTTCAC	
LPR_SY_F4	TCTCATTTCCACCATCATCG	RT-qPCR
LPR_SY_R4	GCCAACGCAATGTTTCACTA	RT-qPCR

RACE, Rapid Amplification of cDNA Ends: TOPO, DNA topoisomerase: PCR, Polymerase Chain Reaction: RT-qPCR, Quantitative reverse transcription PCR: Insitu, Insitu hybridization: dsRNA, double-stranded RNA.

### Phylogenetic analysis

Protein sequences of lipoprotein receptors were obtained from GenBank protein database. These included the vertebrate VLDLRs (Very low density lipoprotein) of *Canis lupus familiaris* (NP_001273907), *Oryctolagus cuniculus* (BAA01874), *Rattus norvegicus* (NP_037287), *Mus musculus* (AAH13622), *Bos taurus* (NP_776914), *Macaca mulatta* (AAR83314), *Pan troglodytes* (XP_520460), *Homo sapiens* (NP_003374); the vertebrate VgRs (Vitellogenin receptors) of *Oncorhynchus mykiss* (CAD10640), *Morone americana* (AAO92396), *Oreochromis aureus* (AAO27569); the vertebrate LDLRs (Low density lipoprotein receptors) of *Mus musculus* (CAA45759), *Homo sapiens* (AAA56833), *Bos taurus* (NP_001160002), *Sus scrofa* (AHF51842), *Chiloscyllium plagiosum* (AAB42184), *Rattus norvegicus* (NP_786938); three lipoprotein receptors (LpRs) of shrimp *Pandalopsis japonica*: LpR1(AHL26189), LpR2A (AHL26190) and LpR2B (AHL26191), and insect LpRs of *Aedes aegypti* (AAQ16410), *Drosophila melanogaster* (NP_733119), *Rhyparobia maderae* (BAE00010), *Locusta migratoria* (CAA03855), *Blattella germanica* (CAL47126), *Bombyx mori* (BAE71406), *Galleria mellonella* (ABF20542); the crustacean VgRs of *Marsupenaeus japonicas* (BAH57291), *Penaeus semisulcatus* (AAL79675), Penaeus monodon (ABW79798), Macrobrachium rosenbergii (ADK55596), *Palaemon carinicauda* (AHB12420), *Pandalopsis japonica* (AHL26192); the insects VgRs of *Drosophila melanogaster* (AAB60217), *Anopheles gambiae* (EAA06264), *Aedes aegypti* (AAK15810), *Solenopsis invicta* (AAP92450), *Periplaneta Americana* (BAC02725), *Rhyparobia maderae* (BAE93218), *Blattella germanica* (CAJ19121). Multiple sequence alignment was performed in BioEdit version 7.2.5 [38] using the clustalW. All the gaps and divergent regions were removed. The aligned protein sequences were exported to Mesquite Version 3.2 [39] and nexus format file was generated. The best-fit model for the protein evolution was obtained from ProtTest V. 3.2 [40] based on the Bayesian Information Criterion (BIC). Phylogenetic analysis was performed with MrBayes v. 3.2 [41] using model (WAG+I+G). To root the tree, the RME2 sequence of *Caenorhabditis elegans* (AAD56241) was used as an outgroup. Two independent Monte Carlo Markov (MCM) chains were executed and sampled every 100 generations for a total of 1000000 generations to approximate the posterior probabilities. The quality of output data was assessed in Tracer v1.6 (http://tree.bio.ed.ac.uk/software/tracer/) and trees were obtained using FigTree v1.4.0 (http://tree.bio.ed.ac.uk/software/figtree).

### In situ hybridization

Single stranded Digoxigenin (DIG) labelled RNA probe of 571 nt was synthesized from cDNA using the DIG RNA labelling kit (Roche). Primers used for the synthesis of sense and antisense RNA probes are listed in Table 1. The concentration and quality of the probes were determined by spot test and spectrometry (Nanodrop ND-1000). *In situ* hybridization was performed in paraffin embedded sections of adult female lice and copepodids as previously described by Dalvin *et al*. [42] and Eichner *et al*. [43] with some modifications. Histoclear (National Diagnostic) was used to deparaffinize the sections and proteinase K treatment was carried out for 18 minutes. Sections were hybridized with DIG-labeled RNA probes (1500 ng/100μl) at 65°C for 20 hr. Afterward, sections were incubated with anti-DIG-alkaline phosphatase Fab fragments (Roche) and visualized using the nitroblue tetrazolium/5-bromo-4-chloro-3-indolyl phosphate (Roche). Sense probe was used as a negative control. Pictures were obtained with an Axio Scope.A1 microscope (Zeiss).

### Immunofluorescence

Immunofluorescence was performed on paraffin-embedded sections of adult female lice. Tissue sections were deparaffinized and rehydrated in a series of graded alcohols. Tissues were blocked with 5% goat serum and 0.1% BSA for 30 minutes. After blocking, sections were incubated with 1:200 dilution of a polyclonal antibody of *Blattella germanica* LpR [32] (a generous gift from Maria-Dolors Piulachs, Institut de Biologia Evolutiva, IBE, Barcelona, Spain). The primary antibodies were detected using goat-anti-rabbit Alexa fluor 488 conjugated secondary antibodies (1: 100, Invitrogen) for 1 hr at room temperature. Sections were washed and mounted with ProLong Antifade mounting media containing DAPI (Life Technologies). Pictures were obtained using a Leica fluorescence microscope.

### Hematoxylin and erythrosine staining

Paraffin-embedded sections of copepodids were stained with hematoxylin and erythrosine according to the procedure as described by Eichner et al. [43]. Briefly, sections were incubated at 65°C for 30 min, dewaxed in histoclear followed by rehydration in a series of graded alcohols. Afterwards, slides were put into distilled water and stained with hematoxylin (Shandon Instant Hematoxylin, Thermo Scientific) for 2.5 min and with 1% erythrosine (Certistain, Merck) for 1.5 min. After staining, slides were washed several times in distilled water and mounted in Histomount (Invitrogen).

### Quantitative reverse transcription PCR (RT-qPCR)

RT-qPCR was carried out on Applied Biosystem 7500 Real-Time PCR system using PowerUp SYBR Green Master Mix (Applied Biosystem) according to the manufacturer's instructions. Primers used in RT-qPCR are listed in Table 1. A standard curve was generated using a two-fold serial dilution (six dilutions) of cDNA to estimate the RT-qPCR assay efficiency. RT-qPCR was performed under the following conditions: 50°C for 2 min, 95°C for 2 min, 40 cycles of 95°C for 15 s and 60°C for 1 min. At the end of the amplification cycles, a melting curve analysis was performed at 60–95°C. As the efficiency of the assay ranged from 95% to 100%, all the assays were carried out simultaneously for *LsLpR* and *ef1α* using the same cDNA and master mix along with two negative controls, a non-template control (NTC) and a reverse transcriptase negative control (-RT). The salmon louse Elongation factor 1 alpha (*ef1α*) was used as a reference gene [44]. All samples were run in duplicate under the following conditions, and Ct (cycle threshold) values were averaged. The expression levels of *LsLpR* was normalized to the expression level of *ef1α*, and the final results were analyzed using 2^-ΔΔCT^ method [45]. Relative expression of *LsLpR* in all RNAi experiments was calculated using the control group as a calibrator. Relative expression levels of *LsLpR* were determined in various developmental stages of salmon louse using chalimus I as a calibrator.

### Production of double-stranded RNA (dsRNA)

Double-stranded RNA (dsRNA) was prepared using Megascript RNAi kit (Ambion). Three different fragments targeting different regions of LsLpR mRNA were amplified by PCR with primers including T7 bacteriophage promoter sequence. The lengths of the dsRNA fragments were, fragment 1; 804 bps (corresponding to nt 1690 to nt 2494 in *LsLpR* mRNA GenBank accession no MF435899), fragment 2; 571 bps (nt 1235 to nt 1804) and fragment 3; 489 bps (nt 2652 to nt 3140). A fragment of 850 bp from cod trypsin (*CPY185*) was used as a control [46]. PCR products were used as templates for the synthesis of sense and antisense RNAs by in vitro transcription using T7 polymerase. Equal volumes of sense and antisense RNAs were pooled, incubated at 75°C for five min and slowly cooled to room temperature. Finally, dsRNAs were purified; concentrations were measured with Nanodrop ND 1000 Spectrophotometer and stored at -20°C until further use.

### RNA interference (RNAi) in nauplii

To knock-down the *LsLpR* in nauplius I larvae, three RNAi experiments were performed separately and each experiment was repeated five times. The first and second RNAi experiments were performed using dsRNA fragment 1 and fragment 2 respectively. The third RNAi experiment was conducted using a combination of dsRNA fragment 1, and 3. Primers used to produce dsRNA used in each experiment are shown in Table 1. All RNAi experiments were performed according to procedure as described in [47]. For all experiments control group was included and animals were treated with dsRNA complementary to *CYP185*. Briefly, egg-strings were gently removed from adult female lice and transferred to flow-through wells. After hatching from the egg-strings, approximately 50 nauplius I larvae were collected for each experimental group in 150 μl of seawater and transferred into Eppendorf tube cap. Nauplii I larvae were incubated overnight (17h) with 1.5 μg of dsRNA. When nauplius I larvae had molted into the nauplius II stage, all animals were transferred into incubators with flow-through sea water supply. *LsLpR* dsRNA-treated animals were inspected daily to detect any abnormal phenotype and the experiment was terminated when the animals reached the copepodid stage 7 days post-hatching (dph). Animals were sampled into RNAlater^TM^ (Ambion) for RT-qPCR analysis.

### Knock-down of LsLpR in pre-adult and adult female lice

The *LsLpR* gene transcript knock-down experiments were done in pre-adult II female lice using previously described three non-overlapping dsRNA fragments. In each experiment, female lice were injected with dsRNA as described in [46] and kept in sea water for 4 hrs. Afterwards, equal numbers (n = 10–13) of dsRNA treated female and untreated male lice were put back on a single fish and a total of three fish were used in each experiment. Each RNAi experiment was terminated when control dsRNA injected female lice had become adults and had produced the second pair of egg-strings. Female lice with or without egg-strings were photographed and examined for changes in gross morphology. Subsequently, the egg-strings were gently removed with forceps, placed into individual hatching incubators and monitored daily to record hatching and developmental progress. Larvae were counted at 9 dph when all control animals were fully developed to copepodids. All lice were sampled and collected in RNAlater (Ambion) for RT-qPCR analysis.

In a single experiment, adult female lice (n = 30) were injected with a combination of *LsLpR* dsRNA fragment 1 and 3. Five injected female lice plus equal amount of untreated male lice were put back per single fish and a total of six fish were used. Same numbers of lice and fish were used for the control group. Lice were recovered after 5, 10 and 15 days post-injection for RT-qPCR analysis.

### Infections of Atlantic salmon with LsLpR knock down copepodids

For infection trials, RNAi was carried out on nauplii I larvae as described above. Five biological parallels each contained approximately 50 nauplii I larvae were treated either with a combination of *LsLpR* dsRNA fragment 1 and 3 or control dsRNA. After that, all the samples were transferred into incubators with flow-through sea water supply. When nauplii molted into copepodids, around 20 copepodids were collected from each parallel for RT-qPCR analysis and remaining copepodids were used for infection of two fish. Each fish in a single fish tank was infected with 60 copepodids according to protocol as described in [43]. The same procedure was followed for the control group. The experiment was terminated when adult female lice of control group produced second pair of egg-strings. All female lice with or without egg-strings were inspected for any gross morphology changes and photographs were taken under microscope. Egg-strings were removed from lice and put into hatching incubators, while female lice were collected for RT-qPCR analysis. Copepodids hatched from these eggs were counted at 9–10 dph.

### Oil Red O staining

Adult female lice were collected directly from the host. Nauplii and copepodids were collected from hatching incubators. Unfertilized eggs from the genital segment and ovaries were dissected from the adult female lice. All the samples were washed three times with cold PBS and fixed in phosphate-buffered 4% paraformaldehyde (pH 7.4). Female lice were fixed overnight while larvae, ovaries and unfertilized eggs were fixed for 2 hrs. Oil Red O stain was performed according to the previously described method [48] with some modifications in the length of time when adult lice were stained. After fixation, all samples were washed three times with ice-cold PBS and resuspended in 60% isopropanol for 10–30 minutes. Larvae and tissue samples were stained with Oil Red O (Sigma-Aldrich) for 0.5 hr while adult lice were stained for 2 hrs. After staining, samples were washed in ice-cold PBS and rinsed with 60% isopropanol. Pictures were obtained with a Leica Model MZ6 stereomicroscope directly or after mounting. For semi-quantification of total neutral lipids, stain was extracted from RNAi copepodids using 200 μl of 100% isopropanol and absorbance were measured at 500 nm in duplicates. Background signal was subtracted using the 100% isopropanol as a background control.

### Protein modelling and bioinformatics analysis

Three-dimensional structure of extra-cellular domains (ligand binding domain from repeat R3-R8 and EGF-precursor domain) of LsLpR protein was modelled using Phyre2 online server [49]. Modelled protein structure was refined using Modrefiner [50]. Calcium ions binding sites were predicted using Raptor X Binding online server (http://raptorx.uchicago.edu/BindingSite/) or COACH for protein-ligand binding site prediction (http://zhanglab.ccmb.med.umich.edu/COACH/) [51]. Various domains of LsLpR protein was predicted using SMART (http://smart.embl-heidelberg.de/) [52]. Signal peptide was predicted using SignalP 4.1 server (http://www.cbs.dtu.dk/services/SignalP/) [53]. Molecular weight and the theoretical isoelectric point of protein was predicted on expasy (http://web.expasy.org/compute_pi/).

## Results

### Sequence analysis of LsLpR

**Fig 1:**
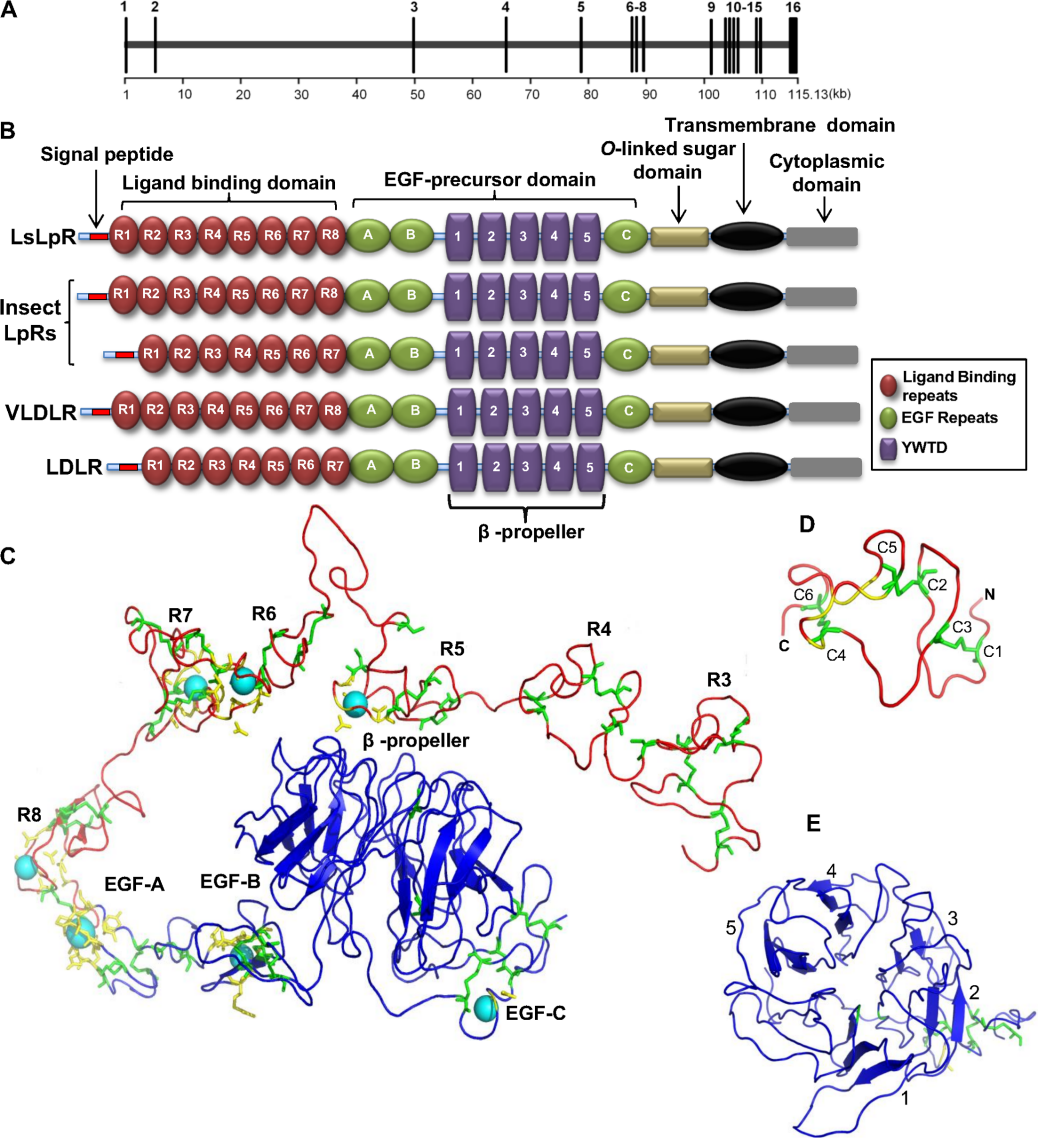
Exon-intron organization and structural analysis of LsLpR. (A) LsLpR gene is composed of 16 exons separated by 15 introns and spanning a genomic region of 115.13 kbp. (B) Domains organization of LsLpR with other members of LDLR family. (C) Modelled structure of extracellular domains of LsLpR using PHYRE protein structure prediction program. Cysteine residues are coloured green, yellow residues provide pocket for calcium ion and bound calcium ions are shown as cyan spheres. (D) Single repeat from ligand binding domain shows the three disulphide bonds (C1-C3, C2-C5 and C4-C6). (E) Top view of β–propeller domain with five F/YWXD motifs.

A full-length cDNA encoding LsLpR was isolated from adult females of *L*. *salmonis*. The full-length transcript was 4007 nucleotides, containing an open reading frame (ORF) of 2859 bp, a 5′-untranslated region (UTR) of 162 bp and a 3’UTR of 986 bp. The ORF of *LsLpR* encodes a putative protein consisting of 952 amino acids, with the signal peptide at position 1–23, the predicted molecular weight (Mw) of 107.04 kDa and the theoretical isoelectric point (pI) of 4.81. The exons-introns analysis revealed that LsLpR gene is composed of 16 exons spanning 115.1 kbp (Fig 1A). The second intron was the largest, spanning about 44.2 kbp.

A BLAST search (http://www.uniprot.org/blast/) against the UniProtKB/Swiss-Prot revealed that LpR of *L*. *salmonis* shared 46% identity (70.8% similarity) with the Lipoprotein receptor 1 from the crustacean *Pandalopsis japonica* and ~48–53% identity (~74–77% similarity) with insect LpRs such as *Locusta migratoria* (migratory locust), *Aedes aegypti* (yellow fever mosquito), *Galleria mellonella* (wax moth), *Bombyx mori* (silk moth), *Blattella germanica* (German cockroach) and *Drosophila melanogaster* (fruit fly). Besides insect LpRs, the *L*. *salmonis* LpR shared ~38–40% identity (~66–68% similarity) with VLDLRs of oviparous vertebrates such as *Salmo salar*, *Danio rerio* (zebrafish), *Gallus gallus* (chicken), *Anas platyrhynchos* (duck) and *Xenopus tropicalis* (frog) and ~36% identity (~67% similarity) with LDLRs of mammals including *Homo sapiens* (human), *Sus scrofa* (wild boar), *Bos taurus* (cattle), *Mus musculus* (mouse) and *Rattus norvegicus* (rat).

### Structural analysis of LsLpR

To analyze the structural and functional domains of LsLpR which are common to members of LDLR superfamily, SMART annotation and multiple protein sequence alignment was carried out. The ligand binding domain (LBD) of LsLpR contained eight cysteine-rich repeats (Fig 1B and S1 Fig). Each cysteine repeat contained six cysteines as shown in modelled extracellular region of LsLpR (green residues in Fig 1C) based on X-ray crystal structure of human LDLR (PDB ID: 1N7D) used as a template [54]. These six cysteines in each repeat formed three pairs of disulphide bonds (C1-C3, C2-C5 and C4-C6) (Fig 1D) which was essential for ligand-receptor interaction [55]. Furthermore, in each repeat a Ca^2+^ binding site was predicted as shown in R5-R8 (Fig 1C and S1 Table) which was considered essential for disulphide formation and correct folding of LpR [56, 57]. Next to the LBD followed the epidermal growth factor (EGF) domain which was important for acid-dependent dissociation of ligands. The EGF domain was composed of three EGF-precursor repeats, and each repeat contained six cysteine residues that made up three pairs of disulphide bonds and a Ca^2+^ binding site (Fig 1C). The EGF domain also contained five F/YWXD tetra-peptide motifs (S2 Fig) required for the formation of a β–propeller structure (Fig 1E) [58]. The predicted *O*-linked sugar domain of LsLpR was composed of a short amino acids sequence consisting of 69 amino acids with phosphorylation sites. The predicted transmembrane domain (TMD) of LsLpR (Fig 1B) contained 23 amino acids helix (AGFMAGVAIGIGAGVILLLFLVL) which was greatly enriched in hydrophobic residues as seen in other LpRs and in other members of LDLR family. TMD-helix acts as membrane anchor [22, 23]. The TMD was followed by the cytoplasmic domain. The cytoplasmic domain of LsLpR carried one copy of NPXY motif (S3 Fig) that is needed for the clathrin-mediated internalization of receptor-ligand complex, and well conserved in LpRs and members of LDLRs family belonging to other species [59]. Presence of several phosphorylation sites in the cytoplasmic domain of LpRs suggested that they are involved in the signal transduction [59]. However, so far there has been no experimental data in insects which support the signal transduction function of LpR [60].

### Phylogenetic analysis

**Fig 2:**
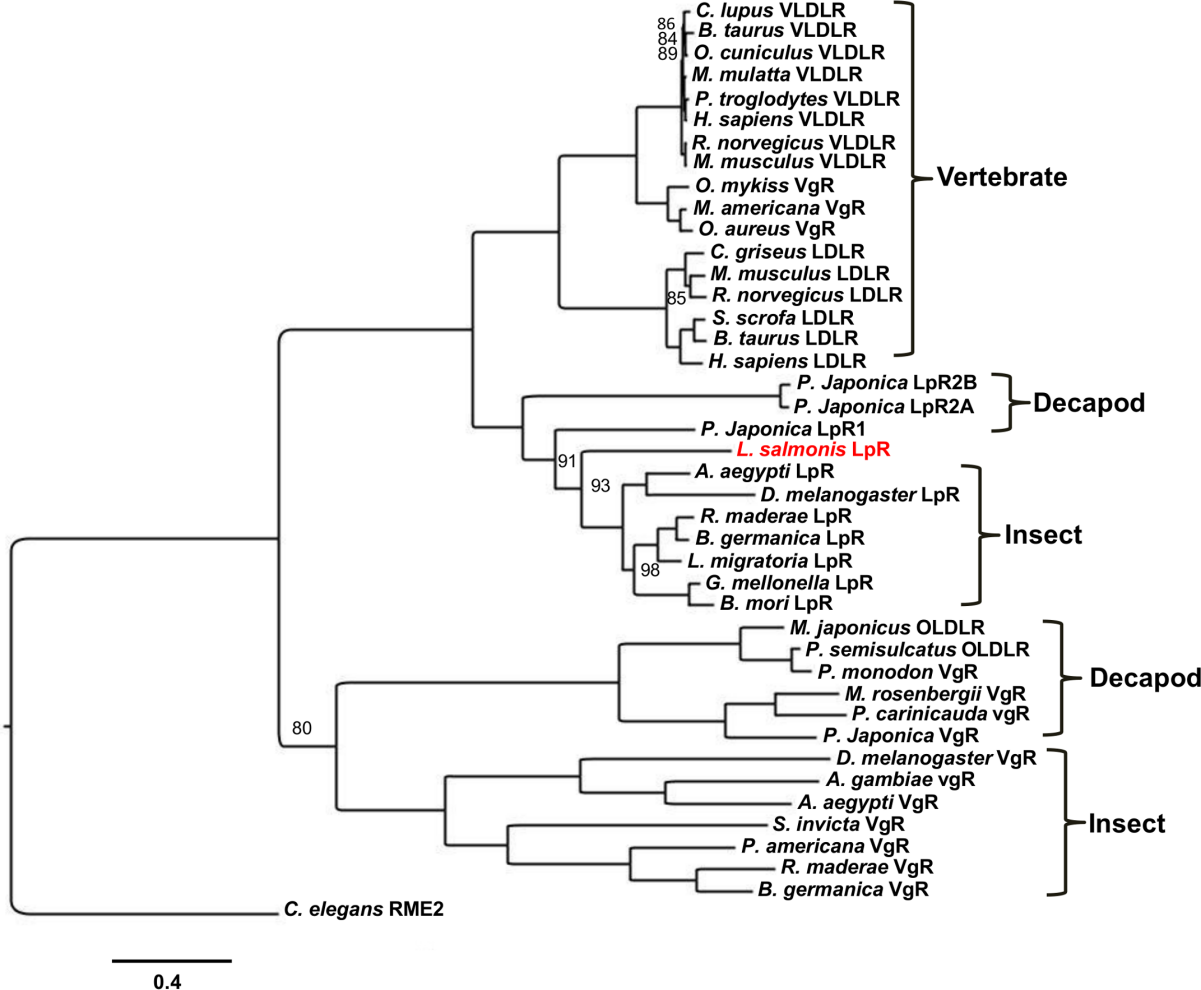
Phylogenetic tree of selected lipoprotein receptors from vertebrates and invertebrates. The tree was generated using Bayesian methods. LpR of L. *salmonis* (LsLpR) is shown in red. The yolk receptor (RME2) of the nematode (*C*. *elegans)* was used as an out-group. The nodes are labelled with posterior probabilities and for clarity only values < 100 are shown. The scale bar represents 0.4 amino acid substitutions per site.

Phylogenetic analysis to reveal evolutionary relationships between LsLpR and lipoprotein receptors from other species is shown in Fig 2. The analysis showed that LsLpR was grouped together with LpRs from decapods and insects. The analysis also revealed that vertebrate lipoprotein receptors (VLDLRs, LDLRs and VgRs) were closely related to each other and closest to decapod/insect LpRs than to VgRs of decapods and insects.

### Expression of LsLpR and distribution of lipids

**Fig 3:**
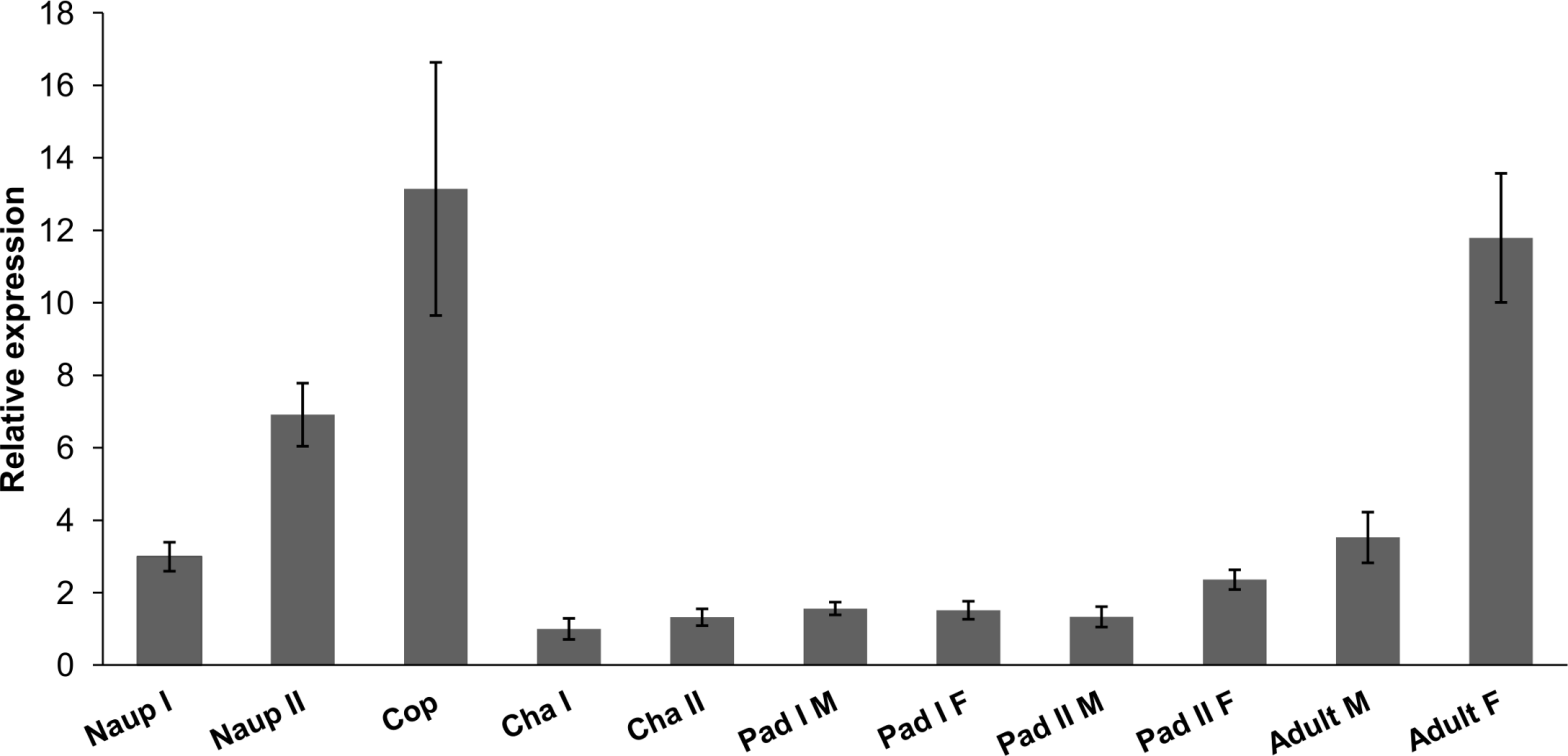
Expression analysis of the *LsLpR* in various developmental stages of the salmon louse. Expression levels of *LsLpR* in chalimus I was set as 1. Error bars represent the standard deviation (n = 5 samples for each stage). Abbreviations: Naup I, Nauplii I: Naup II, Nauplii II: Cop, free-living copepodids: Cha I, Chalimus I: Cha II, Chalimus II: Pad I M, Preadult I male: Pad I F, Preadult I female: Pad II M, preadult II male: Pad II F, Preadult II female.

RT-qPCR analysis was conducted to measure the expression level of *LsLpR* in the different developmental stages of the salmon louse. Expression of *LsLpR* was detected in all the tested developmental stages, with the lowest expression detected in chalimus and pre-adult stages (Fig 3). In larval stages, the lowest expression of *LsLpR* was seen in nauplii I, gradually increased in nauplii II and reached the highest observed level in copepodids (Fig 3). In the mobile stages, the highest *LsLpR* transcript level was detected in the adult female (Fig 3).

**Fig 4:**
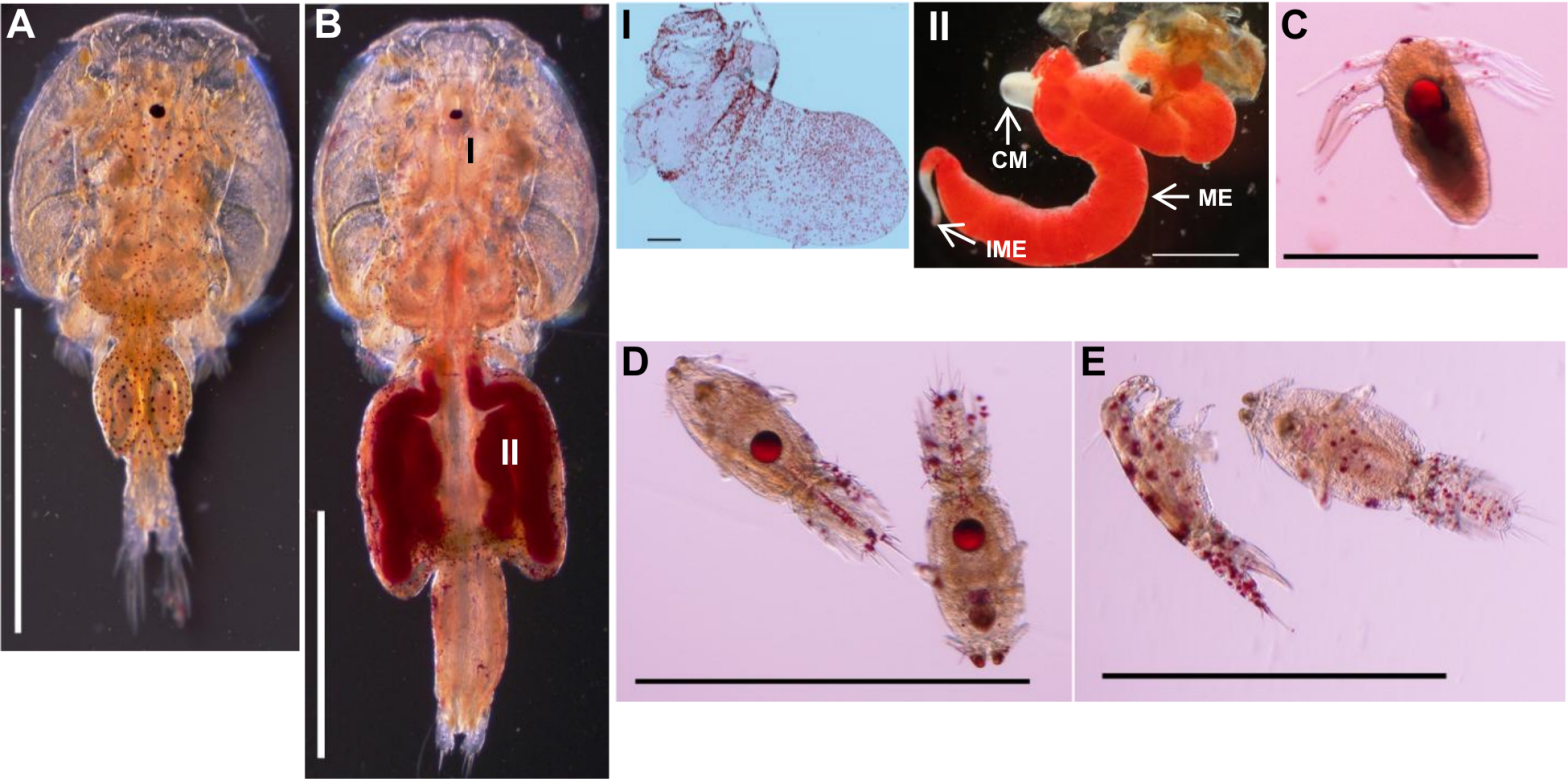
Staining of neutral lipids in salmon lice. Detection of neutral lipids by Oil Red O stain. Adult male (A) and adult female (B). Storage of lipids was detected mainly in mature eggs (II) but also in the ovary (I), of adult female lice. Maternally deposited lipids were found as droplets in the yolk of hatching nauplii (C). A reduction in lipid reserves was noted in copepodids of 7 dph (D) compared to newly hatched nauplii and no lipid droplets were found in copepodids (E) after 10 days of their hatching. Scale bars = (A, B, BII, C-E) 1 mm, (BI) 200 μm. Abbreviations: CM, cement gland; ME, mature eggs; IME, immature eggs.

Neutral lipids were detected in adults and larvae of salmon louse by Oil Red O stain (Fig 4). In adult stages (Fig 4A and 4B), storage of lipids was detected in adult females (Fig 4B), mainly in unfertilized eggs and ovaries (Fig 4I and 4II). In larval stages (Fig 4C–4E), maternally derived lipids were found in the yolk (Fig 4C and 4D), which were utilized by the larvae before their settlement to new host fish (Fig 4E).

### Distribution of LsLpR transcripts in adult female lice and copepodids

**Fig 5:**
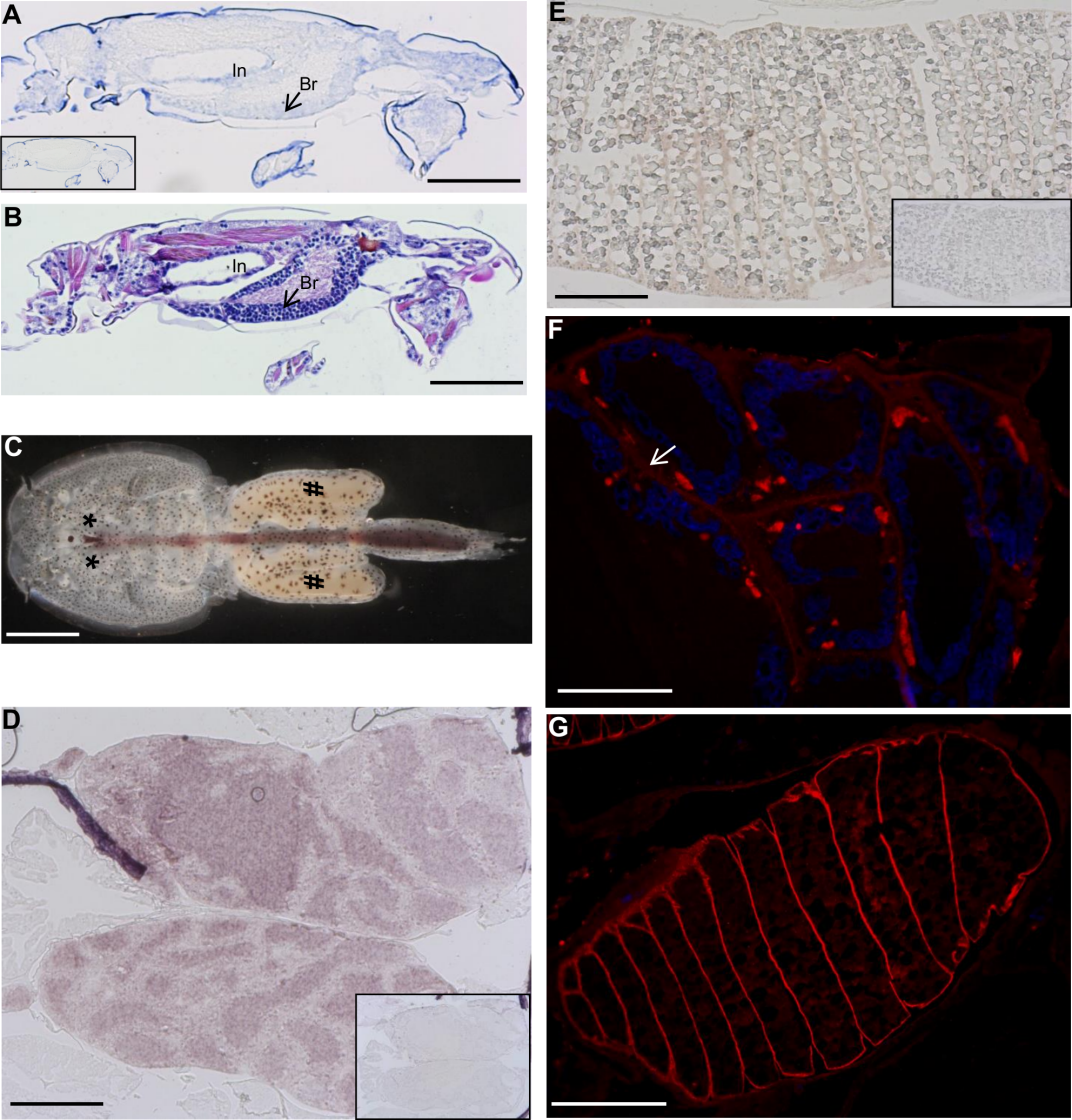
Localization of LsLpR mRNA and protein in the salmon lice. (A), (D) and (E) in situ hybridization. (A) Localization of the *LsLpR* transcripts in the intestine (In) and neuronal somata of the brain (Br) of copepodid. (B) Parallel slide of the copepodid stained with hematoxylin and erythrosine. (C) Dorsal view of an adult female without egg-strings. The asterisks (*) and hashtags (#) indicate the positions of the ovaries in the cephalothorax and mature vitellogenic oocytes in the genital segment of adult female louse respectively. (D) Localization of the *LsLpR* mRNA in the lumen of the ovarian tubules. (E) Localization of the *LsLpR* mRNA in the vitellogenic oocytes in the genital segment. No stain was seen in slides (small inserts) hybridized with sense RNA probe. (F) and (G) immunofluorescence with anti LpR. (F) Distribution of LsLpR protein was found in elongated structures, at the inner side of the tubular membrane (white arrow) together with the nuclei of the oocytes (nuclei were stained blue with DAPI). (G) Distribution of the LsLpR protein in the outer membrane of the vitellogenic oocytes. Scale bars indicate (A-B, E) 200 μm, (C) 1 mm, (D and G) 100 μm, (F) 50 μm.

*In situ* hybridization was performed to examine the distribution of *LsLpR* transcripts. In copepodids, the highest expression of *LsLpR* transcripts was found in the neuronal somata of the brain and the intestine (Fig 5A). In adult female lice (Fig 5C), *LsLpR* transcripts were detected in the lumen of the coiled tubules of the ovaries (Fig 5D) and the outer membranes of the vitellogenic oocytes (Fig 5E). Furthermore, semi quantitative RT-PCR was performed using cDNA of the selected tissues of the adult female lice confirming the results from the *in situ* hybridization (S4 Fig).

### Distribution of LsLpR protein in adult female lice

The presence of LsLpR protein was detected in sections of adult female lice using antibodies raised against LpR of *Blattella germanica* (see Materials and Methods). In ovaries, LsLpR protein was localized in elongated structures, found at the inner side of the tubular membrane together with the oocytic nuclei (Fig 5F). LsLpR was also seen in the outer membrane of the vitellogenic oocytes (Fig 5G), where the *LsLpR* was transcribed (Fig 5E). Moreover, no fluorescence was detected in the control slides treated with secondary antibody only.

### Knockdown of LsLpR in nauplii by RNA interference (RNAi)

**Fig 6:**
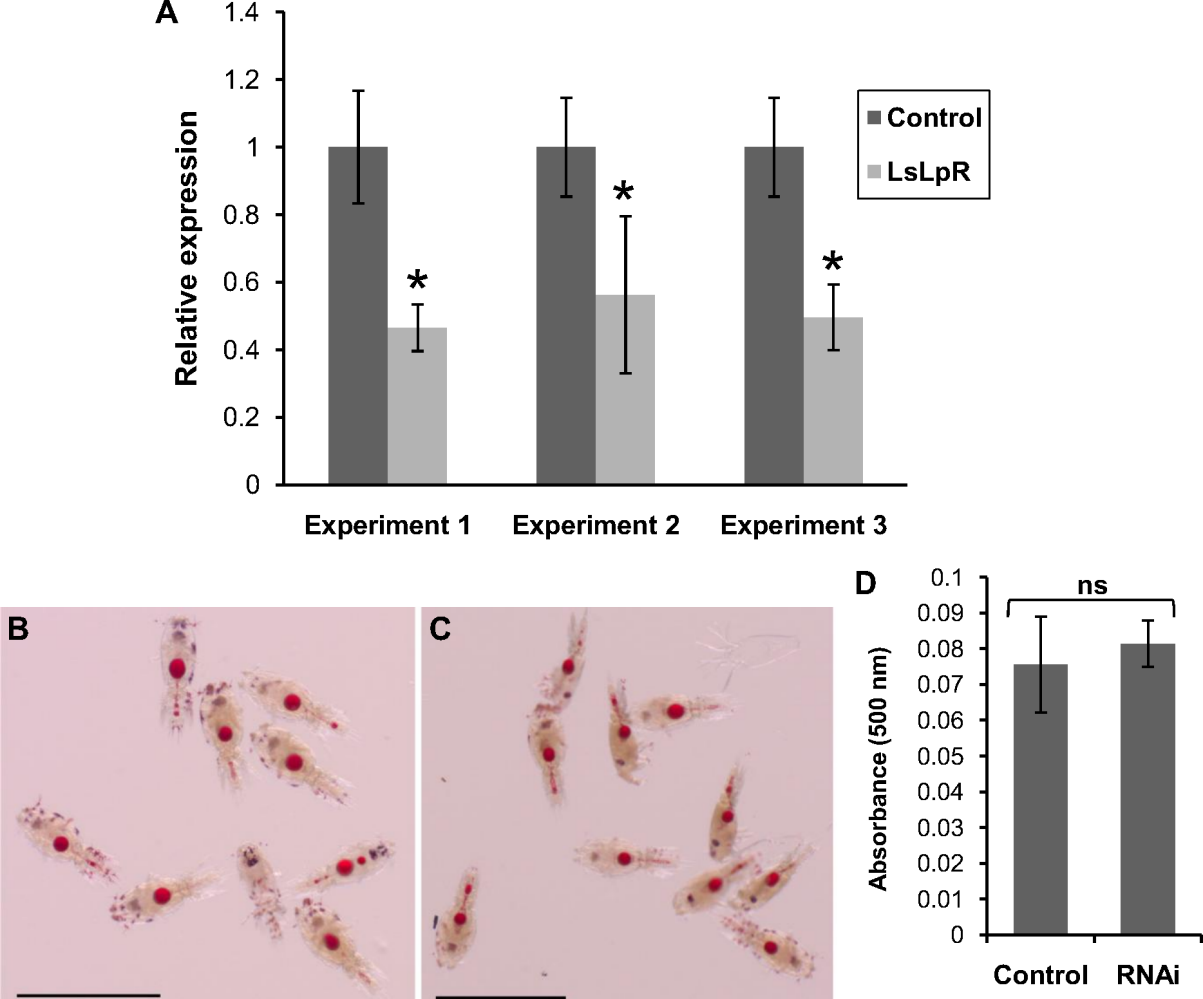
Effect of RNAi on *LsLpR* transcript and lipid levels in copepodids. (A) Relative Expression of *LsLpR* in the copepodids (7 dph) after knock downed in nauplius larva. Error bars show standard deviation. Asterisk represents significant difference (independent-samples T-test, p < 0.05) in mRNA levels of *LsLpR* between the control group (n = 5) and the knock-down group (n = 5). (B-D) Detection of neutral lipids by Oil Red O stain. Lipid contents in the copepodids hatched from *LsLpR* (fragments 1 + 3) (B) and control dsRNAs treated nauplii (C). Semi-quantification of total neutral lipids with Oil Red O stain in copepodids (n = 5, each replicate contains 25 animals) developed from nauplii treated with control and *LsLpR* dsRNAs (fragments 1 + 3) (D). No significant difference (independent-samples T-test, p > 0.05) was found between control group and *LsLpR* dsRNA-treated group. Scale bars = (B-C) 1 mm.

RNAi was induced in nauplius I to access the functional role of *LsLpR* in the larval stages. Three dsRNA fragments (see materials and methods) were produced and utilized in separate RNAi experiments. In the first and second RNAi experiments dsRNA fragment 1 and dsRNA fragment 2 were utilized and transcription of *LsLpR* was decreased by 54% and 44% as compared to control groups respectively (Fig 6A). The third RNAi experiment was conducted using a combination of dsRNA fragment 1, and 3 and *LsLpR* expression was decreased by 50% as compared to control animals (Fig 6A).

However, no gross phenotype or change in survival between control and *LsLpR* dsRNA treated groups was observed. No major difference was found in the lipid staining in the yolk of copepodids developed from nauplii treated with dsRNAs against *LsLpR* and control (Fig 6B–6D).

### Knockdown of LsLpR in Pre-adult II and adult female lice by RNAi

**Fig 7:**
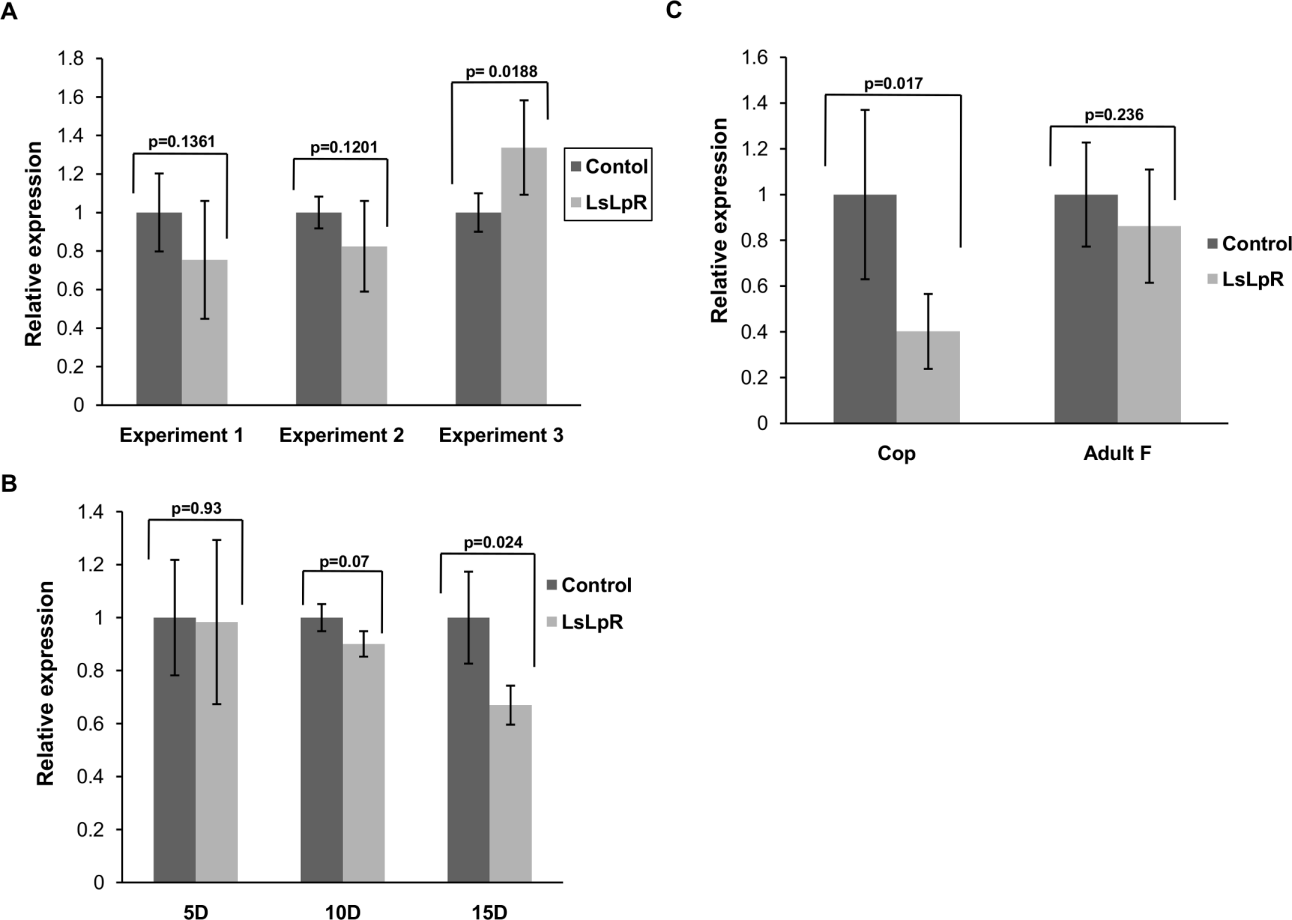
Treatment with dsRNA against *LsLpR*. (A) Relative expression of *LsLpR* in the adult females after injection of dsRNA in pre-adult females (30–32 days post injection). (B) Relative expression of *LsLpR* after injection of dsRNA (fragment 1 + 3) in adult females and measured at days 5, 10 and 15 (post injection). Expression PCR was carried out on 5 female lice from control and knock-down group at each time point. (C) Relative expression of *LsLpR* in copepodids (n = 5 × 20) after knock down (fragments 1 + 3) in nauplii I, assayed before the infection of a host and in adult female lice at the time of termination of the experiment. Error bars show standard deviation and P-values for independent-samples T-test analysis are shown, expression levels of LsLpR in control versus LsLpR dsRNA-treated group.

Three separate RNAi experiments were conducted in pre-adult II female lice and analysed when adult females from control groups had produced the second pair of egg-strings. Eggs from all experimental groups were followed through hatching and development to copepodids. Each experiment was performed with a single dsRNA fragment, or with a combination of two dsRNA fragments (Table 1). The level of *LsLpR* transcripts was measured by RT-qPCR in adult female lice. No significant reduction in mRNA expression levels was observed in lice injected with single dsRNA fragment or a combination of two dsRNA fragments at the time of termination (Fig 7A). Moreover, no significant effect on morphology and survival rate was noted between females injected with *LsLpR* or control dsRNA, but the number of hatched copepodids per adult female was significantly lower (reduced by 72% (p < 0.05, *t* test)) in the *LsLpR*-injected group of experiment 2 compared to the control group (Table 2).

**Table 2:** Summary of the RNAi experiments.

Experiment #	Fragment #	Total Lice injected	Total Lice recovered	No of females carrying Eggs	Average no of copepodids hatched per louse	No of female lice analyzed in RT-qPCR
1	Fragment 1	40	11	11	415±42.5	9
	Control	37	5	5	370.5±55.8	5
2	Fragment 2	30	20	20	145±104	6
	Control	30	18	17	516±166.8	6
3	Fragments 1+ 3	31	23	23	500±105	7
	Control	30	28	28	520±95	6

To see if duration of dsRNA treatment influenced knock down efficiency, adult female lice were injected with LsLpR dsRNA (fragments) and the level of *LsLpR* transcripts was measured at days 5, 10 and 15 (Fig 7B). RT-qPCR results showed that RNAi of *LsLpR* gene could not be detected before day 10. At day 15 transcript levels were reduce by 30% compared to control (p < 0.05, *t* test).

### Infection trial and LsLpR knock down

*LsLpR* knock down (fragments 1 + 3) in nauplii I and level of transcripts were measured by RT-PCR in copepodids (7 dph). In copepodids transcription was decreased by 60% compared to the control group (Fig 7C). Afterwards, Atlantic salmon were infected with the copepodids from the knock down experiment in single fish tanks and maintained on the fish until the lice had developed into adults. Adult female lice were collected and expression of *LsLpR* was measured by RT-PCR. No significant reduction in transcript levels was observed in the adult female lice when compared to control group (Fig 7C). The number of lice recovered from *LsLpR* dsRNA treated group was 30% less than the number of lice recovered from control dsRNA treated group (Table 3). However, no gross abnormal phenotype difference was observed between control and *LsLpR* dsRNA treated groups. Female lice of both groups produced normal egg-strings and equal number of hatched copepodids were found from both groups (Table 3).

**Table 3:** Summary of the infection trial experiment.

Fragment #	No of female lice recovered	No of Male recovered	Female lice which produced egg-strings	Average no of copepodids hatched	No of female lice analyzed in RT-qPCR
Fragments 1+ 3	16, 12	7, 9	16, 12	248±74	5, 5
Control	22, 22	11, 8	22, 17	259±56	5, 5

Nauplii I larvae treated with dsRNA (fragments 1 + 3) from *LsLpR* were sampled as copepodids and used to infect Atlantic salmon, counted as adults (male and female) and if females produced eggstings and finally if the eggs hatched and produced normal copepodid larvae. The numbers represent recovered larvae and adult sea lice from two fish.

## Discussion

In this study, a molecular characterization of the *LpR* from the salmon louse was carried out for the first time. A single copy gene encoding *LsLpR* was identified in the salmon lice genome. Exon-intron organization revealed that LsLpR gene is composed of 16 exons separated by 15 introns. The organization of exons-introns in silkworm, *B*. *mori* for LpR gene has previously been described [31]. The silkworm LpR1 (*Bm*LpR1) was composed of 16 exons interrupted by 15 introns that span about 122 kbp. Whereas, other isoforms such as LpR2, 3 and 4 contained 15 exons separated by 14 introns. The second intron of *Bm*LpR was the largest that span >65 kbp similar to LsLpR where second intron span 44.2 kbp. BLAST searches showed that LsLpR shared the highest amino acid identity and similarity with LpR of decapods and insects. Phylogenetic analysis placed the LsLpR along with other crustacean and insect LpRs and showed that vertebrate VLDLRs/VgRs and LDLRs were closely related to each other and appeared as a sister group of the decapod/insect LpRs. The VgRs of vertebrates did not group together with decapod/insect VgRs indicating that they have evolved independently.

Structural analysis revealed that LpR shared the same structural domains as found in other members of LDLR superfamily. The LBD of LpR usually consist of several cysteine-rich repeats, eight in LsLpR (Fig 1) which was identical to LBD of several insect LpRs such as *B*. *mori*, *L*. *maderae* and *L*. *migratoria*, LpR1 of crustacean *P*. *Japonica* and vertebrates VLDLRs/vitellogenin receptors. However, LBD of some insect LpRs contains seven cysteine-repeats and are structurally identical to LDLRs (Fig 1) [32, 61, 62]. The existance of these repeats in the LBD is imporant for their binding to ligand and the acquisition of cellular lipids but the importance of the numbers of cysteine-repeats in the LBD is not known [62]. LsLpR also contains an EGF-precursor domain which is involved in the acid-dependent displacement of the ligand from the LBD as observed in LDLR-LDL complex at endosomal pH [54, 63, 64]. However, the insect LpR-HDLp complex is not dissociated under an acidic environment, which supports the concept of ligand recycling [65]. The structures of extracellular (LBD and EGF-precursor) domains of human LDLR have been solved by X-ray [54]. The LsLpR shared similar structures when modelled against LDLR. Similar results were found as seen in LsLpR when Locust LpR were modelled against LDLR and it was suggested that despite their high structural similarity, the specificity of both receptors (LDLR and LpR) for their ligands is mutual exclusive [66, 67]. In LsLpR, the EGF-precursor domain followed the 69 residues long *O*-linked sugar domain (OLSD). All insect LpRs contain OLSD, however, the length varies in different insect species [28]. For example, OLSD of *L*. *maderae* is consisting of 70 residues whereas the length of OLSD of *A*. *aegypti* is over 250 residues. Moreover splice variants have been reported that affects this region of OLSD in LpR from other insect species including *B*. *germanica*, A. *aegypti*, *G*. *mellonella* and *B*. *mori* [29, 32, 68, 69]. A single copy of well conserved NPXY internalization motif was found in the cytoplamsic domain of *LsLpR*. The three-dimensional structure prediction and multiple protein sequence alignment both revealed that the sequence of LsLpR contained all structural motifs which are common in LpRs and in other members of LDLR family.

In insects, lipids are transported by the Lp from the fat body to oocytes through receptor mediated endocytosis [18, 33]. Generally, the expression of *LpR* transcripts was observed throughout the ovarian development and increased during vitellogenesis of several insect species including *A*. *aegypti*, *L*. *maderae*, *B*. *germanica*, *S*. *ricini*, *B*. *mori and D*. *melanogaster* [28, 31–34, 62]. Here in salmon lice, high levels of mRNA and protein was found in the ovaries and vitellogenic oocytes of female. Accumulation of neutral lipids was also found in vitellogenic oocytes and ovaries of adult female lice. These results suggest that lipids may be transferred directly from the intestine to growing oocytes and ovaries, where the receptor might be involved in the up-take of lipids to the developing oocytes.

Lipids are the major source of energy for the developing embryos in oviparous animals and 90% of the energy utilized by the developing embryos of *Culex quinquefasciatus* originates from lipids [70]. Similarly, maternally deposited lipids in the larvae of salmon lice are also major source of energy before their settlement to a new host. In larvae, lipids are transported as lipoproteins from their site of storage to different tissues during development. The mechanism of lipoprotein uptake by receptor-mediated endocytosis has been suggested in the fat body tissue of larval and young adult locusts [13, 27]. In salmon lice, the levels of expression and localization of *LsLpR* transcripts in larvae reached its highest level in copepodids where mRNAs of the receptor were found in the intestine and neuronal somata of the brain. These results are in agreement to the expression of *LpR* in the larvae of insect species. In larva of *S*. *ricini*, the expression of *srLpR7-1* was detected in fat body, brain, malpighian tubule, whereas low expression was observed in adult individuals [62]. Similarly in *B*. *mori*, the isoform *LpR-4* was expressed in the brain and central nervous system of larvae along with other developmental stages [31]. In adults of *L*. *migratoria* and *A*. *mellifera*, the expression of LpR was reported in the midgut [27, 35]. The distribution of maternally deposited neutral lipids in the larvae of salmon lice were found in the yolk of hatched nauplii, which were reduced after moulting into copepodids (7 dph) and complete depletion was noted in the aged copepodids (after 10 dph). Notably, the expression of *LsLpR* was highest in the 7 dph copepodids and therefore reflected the transfer of lipids from the yolk to different tissues to secure rapid growth and development.

To further elucidate the function of *LsLpR* in the salmon lice, RNA interference was performed to knock down the *LsLpR* in salmon lice. Three independent RNAi experiments were conducted in the larvae and a significant reduction in *LsLpR* transcripts was noted. However, no change in survival or swimming performance of copepodids were noted and utilization of lipids from yolk were similar in both control and *LsLpR* dsRNA treated groups. It is possible that the levels of knockdown achieved for LsLpR may not be sufficient to disrupt the mobilization of lipids from yolk to other tissues of larvae. Secondly, it is also possible that protein levels were still high within the time frame of these RNAi experiments for the supply of lipids to tissues during larval development. Similar lack of abnormal development was also achieved in the Tsetse fly where the LpR (*GmmLpR*) receptor was significantly knocked down. Here, lipid levels in hemolymph remained unchanged, and oocytes developed normally [71]. Likewise, three independent RNAi experiments were conducted in preadult II female salmon lice. No significant silencing of *LsLpR* was found with any of the three different RNAi fragments and all the adult females produced normal egg-strings. In all RNAi experiments normal development to the copepodid stages was observed; however, in one of the three experiments the numbers of hatched copepodids were reduced from females injected with *LsLpR* (fragment 2) as compared to control. Similar RNAi results were found in *S*. *ricini* [62]. The female pupae of *S*. *ricini* were injected with *LpR* dsRNA along with controls, but no considerable reduction in the mRNA level was found and no abnormalities in ovaries or egg production were noted. Furthermore, RNAi was conducted in *B*. *germanica* and reduction in Lp levels was noted in the ovary but no significant effect on the ovarian development and fertility was noted [32]. Moreover, in the fat body of *B*. *germanica*, the effects of RNAi began to disappear after three days and levels of *LpR* mRNA, and lipophorin contents increased. In salmon lice during infection trial, the *LsLpR* was knocked down in copepodids by 60%. No significant knock-down was observed in adult females that developed from these copepodids (approximately 60 days after infection). Moreover, RNAi experiment in adult females showed that the maximum knock down of *LsLpR* (30%) was only observed at days 15. Hence, it appears that *LsLpR* is difficult to knockdown in adults while in larvae effect of knockdown is not achieved to a level where any obvious abnormal phenotype is observed. Further RNAi studies are needed in the future in different insect and crustacean species to explain the sensitivity of RNAi towards LpRs.

## Supporting information

S1 Fig Sequence alignment of ligand binding domain of LpR, VLDR, LDLR and Vtg receptor. Ligand binding domain of LsLpR, LpR from insects and crustacean (LpRs) and vertebrates and crustacean and VLDR, LDLR and Vtg receptors from vertebrates are aligned. Ligand binding domain of LsLpR is consisting of total eight ligand binding repeats (R1-R8) and each repeat contains six cysteine residues and marked with Asterisks.
(TIFF)

S2 Fig Sequence alignment of EGF-precursor domain of LpRs VLDR, LDLR and Vtg receptors. Sequences of EGF-precursor domain of LsLpRs has been aligned to LpRs from insects and crustacean and vertebrates VLDR, LDLR and Vtg receptors sequences. EGF-precursor domain is consisting of three EGF repeats (EGF-1 to EGF-3) and each repeat contains six cysteine residues which are marked with Asterisks. Five (YWXD (F/Y)) motifs are also present in the EGF-precursor domain which are required for the formation of β–propeller structure.
(TIFF)

S3 Fig Sequence alignment of cytoplasmic domain from LpRs VLDR, LDLR and Vtg receptors. Sequence alignment of cytoplasmic domain of LsLpR, LpRs of insects and crustacean and vertebrates VLDR, LDLR and Vtg receptors. The cytoplasmic domain of LsLpR contain one copy NPXY (X/V) motif which is required for the clathrin-mediated internalization of receptor-ligand complex.
(TIFF)

S4 Fig Expression of *LsLpR* in various tissues of adult female lice. Equal amounts of total RNA from various tissues were reverse transcribed, and RT-PCR was carried out to determine the quantitative variations of *LsLpR* transcripts among samples as analysed on agarose gel. *Ef1a* was used as an internal control. Abbreviations: SQT, sub-cuticular tissue; IN, intestine; OV, Ovaries; OO, Oocytes.
(TIFF)

S1 Table Predicted calcium binding sites in LsLpR. LBD, ligand binding domain: R, repeat: EGF, EGF-precursor domain.
(DOC)
