# Supplementary figures and images for: Molecular characterization of the lipophorin receptor in the crustacean ectoparasite Lepeophtheirus salmonis
Source: PLoS One. 2018 Apr 12;13(4):e0195783. doi: 10.1371/journal.pone.0195783 (PMC5897026; doi:10.1371/journal.pone.0195783)

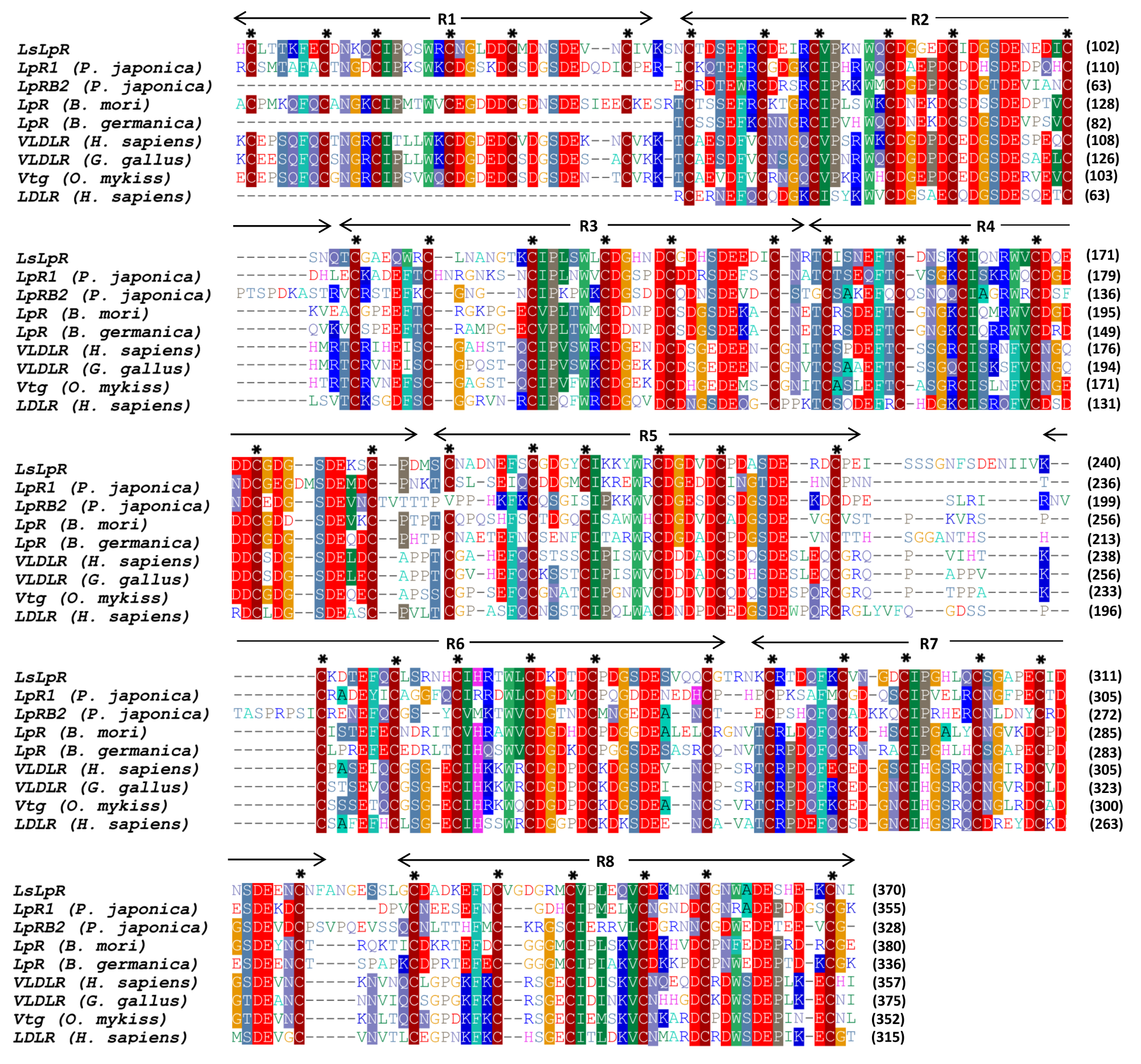

Supplement: S1 Fig — Ligand binding domain of LsLpR, LpR from insects and crustacean (LpRs) and vertebrates and crustacean and VLDR, LDLR and Vtg receptors from vertebrates are aligned. Ligand binding domain of LsLpR is consisting of total eight ligand binding repeats (R1-R8) and each repeat contains six cysteine residues and marked with Asterisks. (TIFF) [file pone.0195783.s001.tiff]

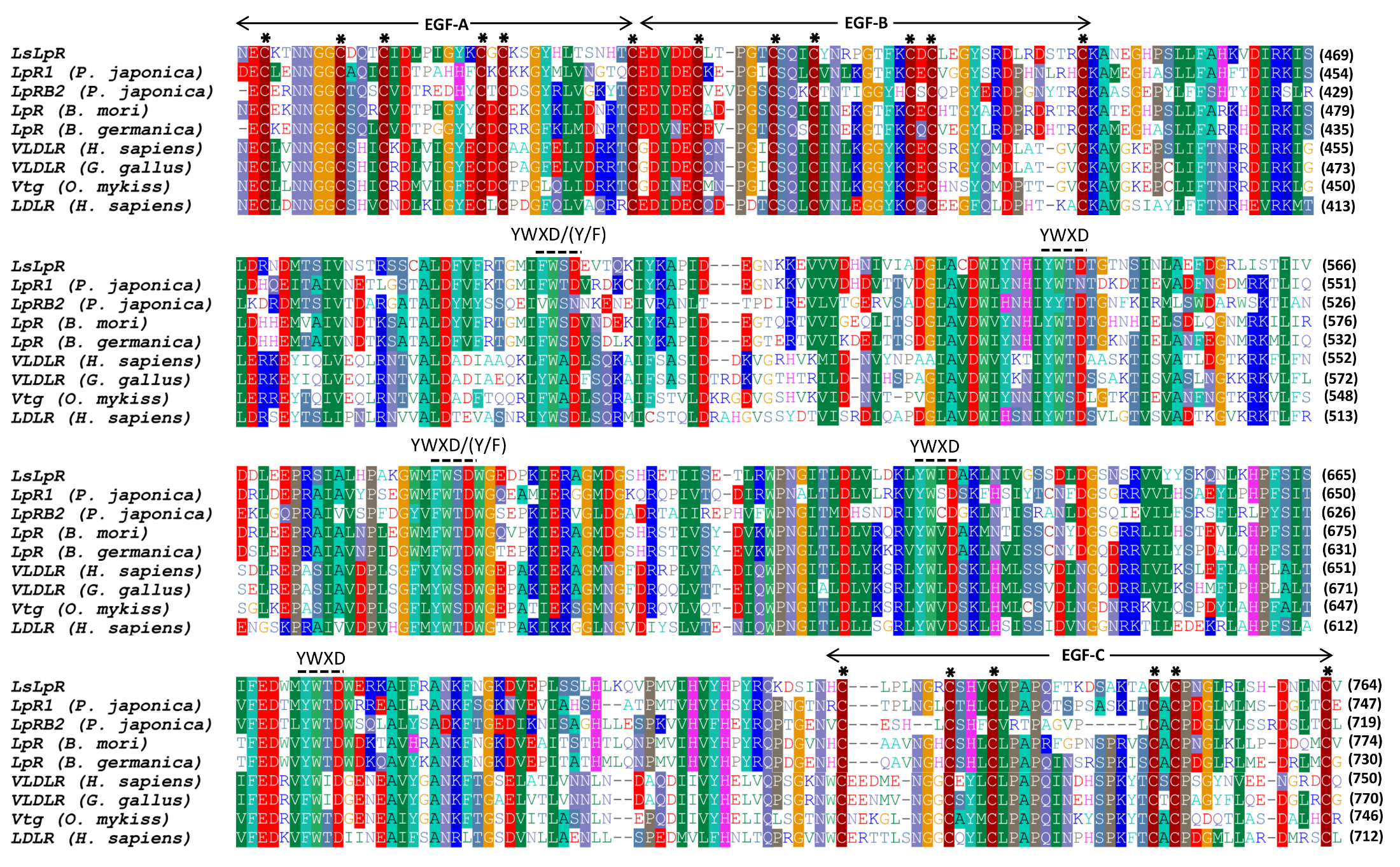

Supplement: S2 Fig — Sequences of EGF-precursor domain of LsLpRs has been aligned to LpRs from insects and crustacean and vertebrates VLDR, LDLR and Vtg receptors sequences. EGF-precursor domain is consisting of three EGF repeats (EGF-1 to EGF-3) and each repeat contains six cysteine residues which are marked with Asterisks. Five (YWXD (F/Y)) motifs are also present in the EGF-precursor domain which are required for the formation of β–propeller structure. (TIFF) [file pone.0195783.s002.tiff]

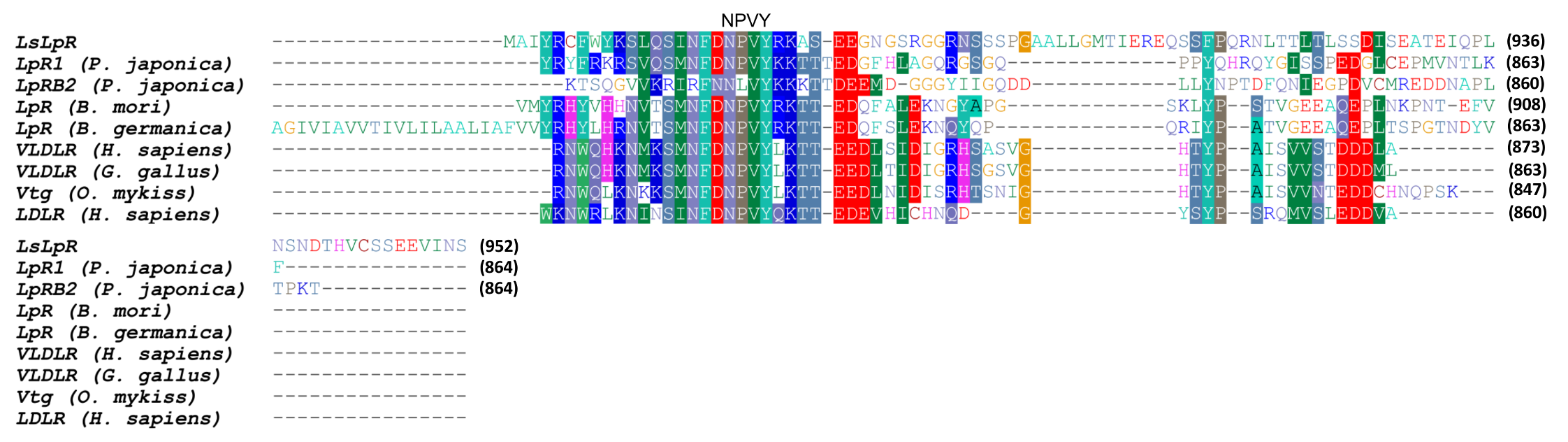

Supplement: S3 Fig — Sequence alignment of cytoplasmic domain of LsLpR, LpRs of insects and crustacean and vertebrates VLDR, LDLR and Vtg receptors. The cytoplasmic domain of LsLpR contain one copy NPXY (X/V) motif which is required for the clathrin-mediated internalization of receptor-ligand complex. (TIFF) [file pone.0195783.s003.tiff]

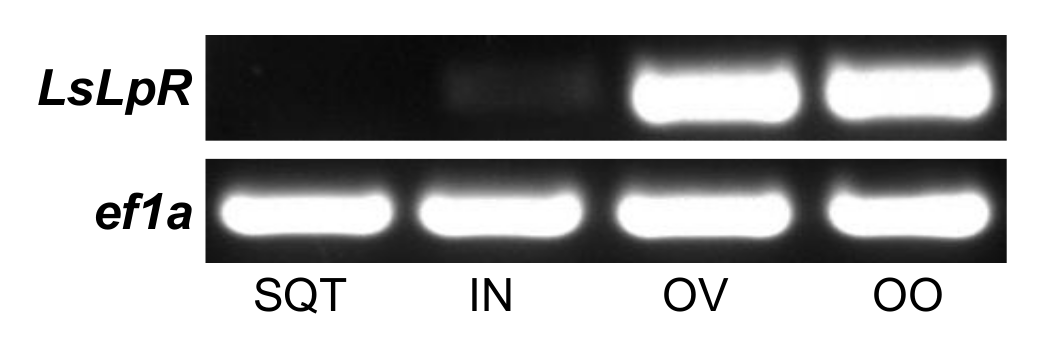

Supplement: S4 Fig — Equal amounts of total RNA from various tissues were reverse transcribed, and RT-PCR was carried out to determine the quantitative variations of LsLpR transcripts among samples as analysed on agarose gel. Ef1a was used as an internal control. Abbreviations: SQT, sub-cuticular tissue; IN, intestine; OV, Ovaries; OO, Oocytes. (TIFF) [file pone.0195783.s004.tiff]
