# Supplementary material for: Molecular characterization of the lipophorin receptor in the crustacean ectoparasite Lepeophtheirus salmonis
Source: PLoS One. 2018 Apr 12;13(4):e0195783. doi: 10.1371/journal.pone.0195783 (PMC5897026; doi:10.1371/journal.pone.0195783)
Supplement: S1 Table — LBD, ligand binding domain: R, repeat: EGF, EGF-precursor domain. (DOC) [file pone.0195783.s005.doc]

| **Binding site** | **Residues** |
| --- | --- |
| LBD (R5) | ASP 106, 108, 110 |
| LBD (R6) | ASP 159, 163, 169, GLU 170 |
| LBD (R7) | GLU 212, ASP 211, LEU 198, GLN 199 |
| LBD (R8) | GLN 246, ASN 253, ASP 259, GLU 260 |
| EGF-A | ILE 267, ASN 268, GLU 269, ASP 283, LEU 284, PRO 285, ILE 286 |
| EGF-B | VAL 307, ASP 308-309, CYS 310, ASN 322, ARG 323 |
| EGF-C | ASN 623 |
